# Diagnosis of Syphilis by the Microscopic Examination of the Blood

**Published:** 1872-02

**Authors:** 


Diagnosis of Syphilis by the Microscopic Examination of the Blood.—In a letter from Vienna, dated January 12th, 1872, to the Boston Medical and Surgical Journal, Dr. James R. Chadwick announces the important discovery by Dr. Lostorfer of what he terms the syphilitic corpuscles in the blood of those affected with syphilis, and that, by a miscroscopic examination of the blood, he can, with certainty, determine the existence or non-existence of syphilis.

We very much regret that space will not permit the publication of the entire letter, but we give such extracts as will present the prominent facts. The following will show the mode of examining the blood, and the results:

"The manner of conducting the researches consisted in taking a drop of blood, placing it immediately between a slide and covering glass, then setting the specimen under cover. A large number of slides were thus prepared, and daily examined microscopically with a No. 10 immersion lens, Hartnack. The first two days, nothing abnormal presented itself; but, on the third, minute shining bodies were discerned, which grew from day to day, until they attained the size of red blood-corpuscles. The shape was, in most instances, round, but modifications of that form were here and there noticed; they were all, however, provided with tapering processes or "sprouts." The number of these corpuscles to be seen at one time within the field of the microscope was variable, amounting in only one specimen to fifty. A month later, these objects were still visible. A solution of sugar, as well as several other solutions, caused them to shrivel up, become, as it were, folded upon themselves, lose their shining aspect, and cease to grow. Cold was also found to affect their development, inasmuch as a temperature below ten degrees Reaumur (fifty-four degrees Farenheit) put a stop to all growth."

After numerous experiments of a character to satisfy himself beyond the possibility of a doubt as to the connection between these bodies and syphilis, Dr. Lostorfer requested Prof. Stricker and Prof. Hebra to put to the test his power of diagnosticating syphilis by the miscroscopic appearances of the blood. They readily consented, and upon various occasions prepared for him numbered slides, with blood of syphilitic and non-syphilitic individuals, and, to their great astonishment, he, unerringly, was able to select from the preparations those where the blood was tainted with syphilis.

The effect produced upon the medical gentlemen assembled at the meeting of the "Gesellschaft der Aerzte," by the announcement of this demonstrated discovery, will be seen by the following sentence from Dr. Chadwick's letter:

"As Dr. Lostorfer ceased speaking, a burst of genuine enthusiasm escaped from the assembled physicians, while Prof. Skoda rose to greet the hero of the evening, as the proclaimer of what was destined to prove one of the most important discoveries of the age. He regretted that it was not customary in Austria to give medals or other rewards of merit in such cases, stating that, had this statement been made in Paris, he would have been crowned, as he deserved."

As above stated, Dr. Lostorfer gives the name of syphilitic corpuscles to these bodies, and is of the opinion that they originate in one of two ways: "either from germs always present in the blood, but stimulated to growth by the syphilitic virus alone, or from such introduced bodily into the blood at the time of infection."

